# Performance-Enhanced Triboelectric Nanogenerator Based on the Double-Layered Electrode Effect

**DOI:** 10.3390/polym12122854

**Published:** 2020-11-29

**Authors:** Seungju Jo, Inkyum Kim, Nagabandi Jayababu, Daewon Kim

**Affiliations:** Department of Electronic Engineering, Institute for Wearable Convergence Electronics, Kyung Hee University, 1732 Deogyeong-daero, Giheung-gu, Yongin 17104, Korea; joseungju@khu.ac.kr (S.J.); inkyum.kim@khu.ac.kr (I.K.); nagabandi.jay@khu.ac.kr (N.J.)

**Keywords:** triboelectric, water-assisted oxidation, sputtering, double-layered effect

## Abstract

Recently, studies on enhancing the performance of triboelectric nanogenerators (TENGs) by forming nanostructures at the contacting interface have been actively reported. In this study, a double-layered bottom electrode TENG (DE-TENG) was successfully fabricated using a metal deposition layer after the water-assisted oxidation (WAO) process. As previously reported, the WAO process for the enhancement of electrical performance increases the effective contact area with an inherent surface oxidation layer (Al_2_O_3_). As a new approach for modifying deficiencies in the WAO process, a metal deposition onto the oxidation layer was successfully developed with increased device output performance by restoring the surface conductivity. The proposed metal–dielectric–metal sandwich-structured DE-TENG generated approximately twice the electrical output generated by the WAO process alone (WAO-TENG). This dramatically improved electrical output was proven by a theoretical demonstration based on a double capacitance structure. In addition, the double capacitance structure was confirmed with the aid of a field emission scanning electron microscope. The optimal point at which the DE-TENG generates the highest electrical outputs was observed at a specific Cu layer sputtering time. The exceptional durability of the DE-TENG was proved by the 1 h endurance test under various relative humidity conditions. The potential of a self-powered force sensor using this DE-TENG is demonstrated, having a comparably high sensitivity of 0.82 V/N. Considering its structure, increased electrical energy, easy fabrication, and its durability, this novel DE-TENG is a promising candidate for the self-powered energy harvesting technology in our near future.

## 1. Introduction

Energy harvesting, a process that derives power from external sources such as solar radiation [1,2,3], thermal energy [4,5], wind energy [6,7,8] and kinetic energy [9,10], has attracted a great deal of attention due to its numerous advantages that offer a promising path toward energy alternatives in the future. A variety of methods have been demonstrated that convert mechanical energy into electrical energy using different mechanisms, such as piezoelectricity [11,12], electromagnetics [13], and triboelectricity. Among the various technologies, the triboelectric nanogenerator (TENG), based on the integration of triboelectrification and electrostatic induction, was developed for the efficient conversion of mechanical energy into electrical energy [14,15,16]. This TENG operates using the contact of two different materials with opposing tendencies of electron affinity. More specifically, triboelectrification provides oppositely polarized charges on each contact material’s surface, following which, electrostatic induction drives the transformation of mechanical energy into electrical energy. In addition to this simple operating mode, the TENG has many advantages, including cost-effectiveness, a low weight, simple fabrication, and a high output voltage at a very low frequency [17,18]. Therefore, this is a promising technology in the area of mechanical energy harvesting. In addition, applying various technologies to TENG devices opens new possibilities for the use of TENGs in numerous applications [19,20,21].

In recent years, the tremendous efforts to enhance the electrical properties of TENGs have attracted significant attention. Therefore, it is important to increase triboelectric charge density with the proper selection of contact materials that have largely different triboelectric charging polarities. The enhancement of effective contact area, along with the increase in the surface charge density, is the key to improving the performance of TENGs [22,23,24,25,26]. Recently, a few attempts have been made to address this issue, but these have not fully succeeded; the actual issue, related to the effective enhancement of surface charge density, remains unresolved [27,28,29,30,31,32,33,34,35,36].

In this study, a novel DE-TENG with a nanomorphology-structured metal layer and double-capacitance-based structure was successfully developed, displaying advanced electrical performance. Aluminum (Al) is a cost-effective material, and the water-assisted oxidation (WAO) process offers a simple and rapid route to create nanostructures on a metal surface. Using the additional deposition after the WAO process, this simplified structure and surface modification, with a double capacitance structure, are the keys to this electrical enhancement. Based on the effect of the double electrode layer for restoring the reduced conductivity of the oxidation layer, the electrical output of the DE-TENG was about two times higher than that of the WAO-TENG. Owing to the tremendous enhancements in the performance of TENG, the technology based on the double electrode effect can be widely applied to various applications.

To demonstrate the enhanced electrical performance of the DE-TENG, a charging test was conducted using a 0.1 μF capacitor, which is a much higher capacity than WAO-TENG. Furthermore, 25 commercial LED bulbs can be simultaneously lit up with the power generated by the DE-TENG, whereas bare TENG and WAO-TENG can light up 9 and 17 LED bulbs, respectively. We proved that the DE-TENG has high applicability with its enhanced power sensors compared to the pristine TENG (without the double electrode layer). With the reliable stability of the endurance test under various levels of relative humidity, the applicability of sensitive force sensors with a linear fit was effectively proven. On the basis of the above work, the additional electrode deposition following the WAO process is advantageous for the enhanced performance of the DE-TENG in terms of its novel structure and simple fabrication.

## 2. Experimental Section

### 2.1. Fabrication of the Double Electrode-Layered TENG

**Figure 1:**
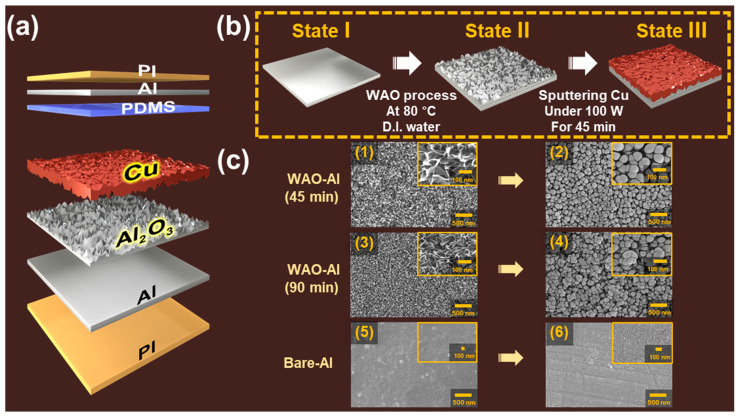
(**a**) Schematic illustration of the double-layered electrode triboelectric nanogenerator (DE-TENG); (**b**) the fabrication process of the DE-TENG (water-assisted oxidation process and additional deposition layer with sputtering) and (**c**) the field emission scanning electron microscope (FE-SEM) images (WAO-Al treated for (**c1**) 45 min and (**c3**) 90 min, Cu-sputtered onto the layer of WAO-Al for (**c2**) 45 min and (**c4**) 90 min, (**c5**) Bare-Al and (**c6**) Cu sputtered onto the Bare-Al).

A DE-TENG was fabricated with the combination of an Al layer (WAO process, Smato, Seoul, Korea) and Cu layer (sputtered, TAEWON SCIENTIFIC, Seoul, Korea), along with Polydimethylsiloxane (PDMS, OMNISCIENCE, Gyeonggi-do, Korea) and bare Al (Smato, Seoul, Korea) (Figure 1a). A typical synthesis process was performed as follows: firstly, commercially available Al tape was oxidized at 80 °C in deionized water for 45 and 90 min to obtain the oxidized Al layers. Subsequently, the Cu layer was coated on to this oxidized Al layer by sputtering. The power level of the sputtering was fixed at 100 W and the working time was varied (15, 30, 45, and 60 min) during the process. Finally, the device was constructed by means of the systematic assembly of an oxidized Al layer, Cu layer, PDMS, and bare Al with polyimide (PI, YOUNGWOO TRADING, Gyeonggi-do, Korea) as a substrate (Figure 1a).

### 2.2. Characterization and Electrical Measurements

The surface morphologies and chemical composition of the DE-TENG were analyzed using a high-resolution field emission scanning electron microscope (HR FE-SEM, Carl Zeiss MERLIN, Oberkochen, Germany) and energy dispersive spectroscopy (EDS, Carl Zeiss MERLIN, Oberkochen, Germany), respectively. A Keithley 6514 electrometer (OH, USA) was used to measure the open-circuit voltage and short-circuit current (*V*_oc_ and *I*_sc_) of the DE-TENG. An electrodynamic shaker (Labworks Inc. LW139.138-40, Costa Mesa, CA, USA) was employed to generate contact-separation processes by converting the electrical signal into linear mechanical movement.

## 3. Results and Discussion

As shown in Figure 1a, the novel DE-TENG is composed of a double-layered bottom electrode on a PI substrate. The top friction layer consists of PDMS-coated Al, while the bottom part contains the WAO-Al layer, which is completely covered by the Cu layer. Figure 1b depicts the detailed process of fabricating the double-layered bottom electrode. A bare Al sheet can be seen in State I, and State II represents the WAO-processed Al. As shown in the figure, we confirmed that the nanograss-like structures were uniformly formed on the surface of Al, which had arisen from the formation of the Al_2_O_3_ layer. The WAO process can not only provide a dielectric layer (Al_2_O_3_) but a nanograss-structured layer on the surface, which enhances the output performance of the fabricated device. In State III, the Cu was deposited on Al_2_O_3_ via sputtering, which resulted in the formation of a final double electrode layer. As a result, the additional deposition of Cu onto the Al_2_O_3_ layer provided the enhanced effective surface area with a nanostructured morphology as well as the improved surface conductivity of the electrode. Therefore, the simplified structure and surface modification based on the WAO process following Cu deposition is the key to improving the electrical performance. This process neither requires any sophisticated equipment nor much time for the fabrication process.

Figure 1c demonstrates the surface morphologies before and after the sputtering process. The typical surface morphologies of the WAO-Al with different WAO process times (45 and 90 min) were investigated by means of a field emission scanning electron microscope (FE-SEM) and are shown in Figure 1(c1,c3). The longer WAO process (90 min) resulted in denser grass structures. Figure 1(c5) shows the flat surface of bare Al. Figure 1(c2,c4,c6) demonstrates the surface morphologies of the same samples after the sputtering process. After the deposition of Cu on the surface of WAO-Al, the nanograss-like morphology of WAO-Al (Figure 1(c1,c3)) was transformed into pebble-like structures with decreased roughness of DE-Al (Figure 1(c2,c4)). By depositing Cu onto the Al_2_O_3_ layer, the nanomorphology-structured metal layer was successfully created on the surface, which was the already-increased effective contact area driven by the WAO process. This device can promote the enhancement in surface conductivity, along with the development of new structures. The actual optical images of WAO-Al and DE-Al are included in Figure S1. Figure 1(c6) shows the surface morphology of bare Al after Cu deposition, which indicates a flat surface without any specific structures. Therefore, the final effective contact area and surface charge density of DE-TENG is much higher than Cu sputtered onto bare Al. Moreover, the enhanced surface conductivity with metal deposition (DE-TENG) can possibly improve the electrical performance of the already-developed WAO-TENG.

**Figure 2:**
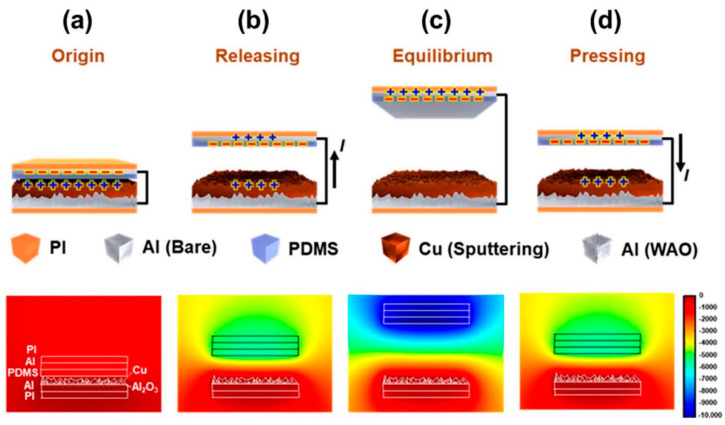
The working mechanism and potential difference from the COMSOL simulation results of the DE-TENG. (**a**) the origin state, (**b**) the releasing state, (**c**) the equilibrium state, and (**d**) the pressing state.

The working mechanism of the DE-TENG and the COMSOL simulation of each state is shown in Figure 2. The DE-TENG generated electricity is based on the coupling of the triboelectric effect and electrostatic induction. The output of the DE-TENG was obtained during the contact-separation of two friction layers due to their relative triboelectric series. In the original state, the top friction layer (dielectric material) was in contact with the bottom layer (electrode) (Figure 2a). In this state, the PDMS surface is negatively charged and the surface of the double electrode layer is positively charged without applying any mechanical force. In Figure 2b, when the top and bottom friction layers are separated by an external impact, a higher potential is induced on the bottom layer’s electrode than on the top. Thus, the current flows from the bottom to the top electrode through an external load by producing an electrical potential difference. Figure 2c represents the equilibrium state where the two layers are fully separated, so that there is no charge flow. In this state, the potential difference and the amount of transferred charges reached the maximum value. In Figure 2d, the distance between the two layers is closer, and the current flows from the top electrode to the bottom in an opposite direction to that shown in Figure 2b.

**Figure 3:**
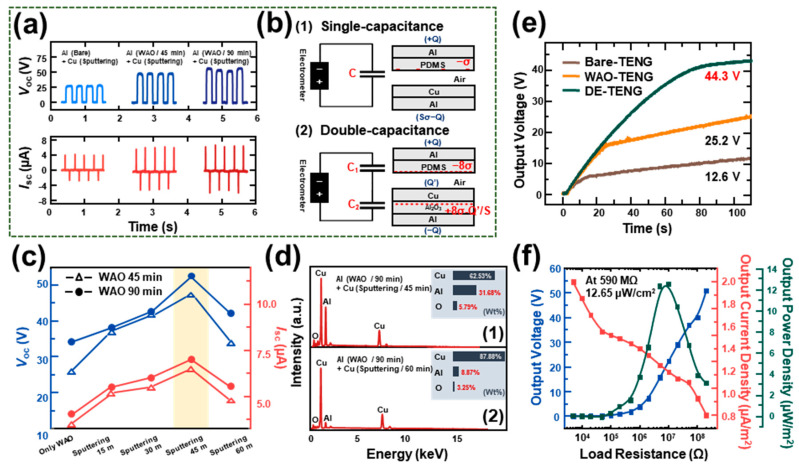
The electrical output from the DE-TENG compared to the pristine TENG: (**a**) The electrical output tendency (*V*_oc_ and *I*_sc_) and (**b**) the schematic structure of (**b1**) single and (**b2**) double capacitance; (**c**) the electrical output tendency of WAO-TENG and DE-TENG under various sputtering times; (**d**) the EDAX data for the DE-TENG (sputtering under (**d1**) 45 and (**d2**) 60 min); (**e**) output voltage from charging of a 0.1 μF capacitor (bare-TENG, WAO-TENG, and DE-TENG); (**f**) output power density, output voltage, and output current density along the load resistances.

Figure 3a indicates the enhanced electrical performance (*V*_oc_ and *I*_sc_) of the device with double capacitance (Cu layer sputtered on the WAO-Al) compared to that with single capacitance (Cu layer sputtered on the bare Al). To demonstrate the effect of double capacitance, Figure 3(b1) and Figure 3(b2) show the structure for single and double capacitance, respectively. In Figure 3(b1), the device contains only one dielectric layer (PDMS) while the device in Figure 3(b2) contains two dielectric layers (PDMS and Al_2_O_3_). The DE-TENG structure with double capacitance inherent to the Al_2_O_3_ layer can be considered a new model, as shown in Figure 3(b2) [37].

Models of single- and double-capacitance-structured TENGs can be simply expressed by the following equations with the amount of charge on each electrode (*Q*), the surface charge density (*σ*), the area size (*S*) of metals, the permittivity of the dielectric (dielectric constant, *ε*), and the thickness of the dielectric layer (*d*). Based on these parameters, the electrical field strength of the above two models (Figure 3(b1) and Figure 3(b2)) was derived by Gauss’s theorem.

The electric field strength at each region for the single capacitance model (Figure 3(b1)) is given by:

(1) $$Inside\;PDMS:E_{PDMS}=\frac{Q}{S\varepsilon_{0}\varepsilon_{PDMS}}$$

(2) $$Inside\;the\;Air\;gap:E_{Air}=\frac{\frac{Q}{S}-\sigma }{\varepsilon_{0}}$$

The voltage between the two electrodes can be given by:

(3) $$V=E_{PDMS}d_{PDMS}+E_{Air}d_{Air}$$

Substituting Equations (1) and (2) into Equation (3), the *V-Q-x* relationship for the single capacitance is given by:

(4) $$V=\frac{Q}{S\varepsilon_{0}}\left(\frac{d_{PDMS}}{\varepsilon_{PDMS}}+d_{Air}\right)-\frac{\sigma d_{Air}}{\varepsilon_{0}}$$

At the open-circuit (OC) condition, there is no charge transfer (*Q*). Therefore, the open-circuit voltage of single capacitance is given by:

(5) $$V_{oc}=-\frac{\sigma d_{Air}}{\varepsilon_{0}}$$

Similarly, the model for double capacitance is shown in Figure 3(b2). In this structure, the surface charge density is much larger than the previous case with the increased effective contact area by the effect of the Al_2_O_3_ layer. In contact-separation mode, there are remaining charges (*Q’*) on the Cu layer. Therefore, the electric field strength at each region in the model for double capacitance is given by:

(6) $$Inside\;PDMS:E_{PDMS}=\frac{\left(Q-Q^{\prime }\right)}{2S\varepsilon_{0}\varepsilon_{PDMS}}+\frac{4\sigma }{\varepsilon_{0}\varepsilon_{PDMS}}$$

(7) $$Inside\;the\;Air\;gap:E_{Air}=\frac{\left(Q-Q^{\prime }\right)}{2S\varepsilon_{0}}-\frac{4\sigma }{\varepsilon_{0}}$$

(8) $$Inside\;Al_{2}O_{3}:E_{Al_{2}O_{3}}=\frac{Q}{4S\varepsilon_{0}\varepsilon_{Al_{2}O_{3}}}+\frac{4\sigma }{\varepsilon_{0}\varepsilon_{Al_{2}O_{3}}}$$

The voltage between the two electrodes can be given by:

(9) $$V=E_{PDMS}d_{PDMS}+E_{Air}d_{Air}+E_{Al_{2}O_{3}}d_{Al_{2}O_{3}}$$

After substituting Equations (6)–(8) into Equation (9), the *V-Q-x* relationship for the double capacitance is given by:

(10) $$V=\frac{1}{2S\varepsilon_{0}}\left(\frac{(Q-Q^{\prime })d_{PDMS}}{\varepsilon_{PDMS}}+\frac{Qd_{Al_{2}O_{3}}}{2\varepsilon_{Al_{2}O_{3}}}+(Q-Q^{\prime })d_{Air}\right)+\left(\frac{4\sigma d_{PDMS}}{\varepsilon_{0}\varepsilon_{PDMS}}+\frac{4\sigma d_{Al_{2}O_{3}}}{\varepsilon_{0}\varepsilon_{Al_{2}O_{3}}}-\frac{4\sigma d_{Air}}{\varepsilon_{0}}\right)$$

At the OC condition, the open-circuit voltage of double capacitance is given by:

(11) $$V_{oc}=\frac{4\sigma d_{PDMS}}{\varepsilon_{0}\varepsilon_{PDMS}}+\frac{4\sigma d_{Al_{2}O_{3}}}{\varepsilon_{0}\varepsilon_{Al_{2}O_{3}}}-\frac{4\sigma d_{Air}}{\varepsilon_{0}}$$

From Figure 1c, in comparison to the nanomorphology-structured surface of DE-TENG (Cu sputtered on WAO-Al) (Figure 1(c2,c4)), the surface of Cu sputtered on bare Al (Figure 1(c6)) is flat and without nanostructure. With the enlarged effective contact area, the surface charge density for DE-TENG is much higher than that of general TENG. Compared to each open-circuit voltage for single and double capacitance (Equation (5) and Equation (11)), the final value of double capacitance (followed by the increased surface charge density and effective contact area with the existence of the Al_2_O_3_ layer) is twice as high as that of the single capacitance. The experimental result of electrical outputs (Figure 3a) was exactly consistent with this theoretical demonstration. The metal–dielectric–metal sandwich-structural DE-TENG with the Al_2_O_3_ layer has a double capacitance model, compared to the general TENG with its single capacitance structure. From this process, the effect of the WAO process with the existence of the Al_2_O_3_ layer can be proved to enhance the performance of the TENG. Therefore, it is essential to analyze the electrical output of DE-TENG specifically, because the advantage of the WAO process in enhancing the TENG performance was already observed in a previous study. Figure 3c represents the effect of Cu deposition after the WAO process (DE-TENG) with a comparison to WAO-TENG. From the figure, the electrical output of the DE-TENG is higher than WAO-TENG owing to the greater positive charges of the Cu- than the Al-based triboelectric series. The reduced conductivity during the WAO process can be restored by metal deposition onto the Al_2_O_3_ layer. Moreover, the enhanced electrical output can be attributed to the double-capacitance metal–dielectric–metal structure. In these respects, the effect of the additional metal deposition after the WAO process is effectively proved by the nanomorphology-structured metal layer’s improved electrical output. Figure 3d demonstrates the optimum metal deposition condition for specific analysis in DE-TENG. The results of the electrical measurements obtained with the various sputtering times suggest the existence of an optimal point in the *V*_oc_ and *I*_sc_ for the DE-TENG. The sputtering time of the DE-TENG spanned 15 to 60 min, while the corresponding *V*_oc_ gradually increased until 45 min and then decreased. Therefore, 45 min was identified as the optimal sputtering time. Furthermore, the electrical output of the samples treated with the WAO process for 45 min had a similar tendency as the samples treated with the WAO process for 90 min. However, the electrical output of a 90 min-treated device was only slightly higher than the electrical output of a 45 min-treated device. To demonstrate the existence of an optimal sputtering time, the EDS spectra of DE-TENG obtained for sputtering times of 45 and 60 min are shown in Figure 3(d1) and Figure 3(d2), respectively. Longer deposition times after the WAO process thicken the additional metal layer. Thus, the surface charge density and the effective charge area increased with 60 min sputtering, which resulted in greater electrical output than the other methods. However, the surface roughness decreased with the deposition time. In Figure 3(d2), the ratio of Cu and Al is greater than in Figure 3(d1), which is due to the deposition of more Cu for longer sputtering sessions. At 60 min of sputtering, the surface became flat and the roughness decreased (Figure S2), which ultimately resulted in the decrease in the effective contact area and electrical output. At more than 60 min for the sputtering process, it is thought that the final surface is similar to bulk Cu due to the formation of a smooth, film-like surface. The charging capacities of various systems (bare TENG, WAO-TENG, DE-TENG) were tested by charging a 0.1 μF capacitor, and the obtained results are shown in Figure 3e. The DE-TENG charged the capacitor to 44.3 V, the WAO-TENG to 25.2 V, and the bare TENG to 12.6 V within 110 s. These results show the advanced electrical performance of the DE-TENG. We also noticed that the electrical performance of the DE-TENG was about twice that of the WAO-TENG. This is because the surface conductivity can be increased by a nanomorphology-structured metal layer based on the metal–dielectric–metal sandwich-structural double capacitance model. Figure 3f demonstrates the variation in the output voltage, output current density, and output power density with varying external load. The maximum output power density of the DE-TENG (12.65 μW/m^2^) was observed when the external resistance reached 590 MΩ. The output current density decreased with the increasing load resistance, while the output voltage showed the opposite trend.

**Figure 4:**
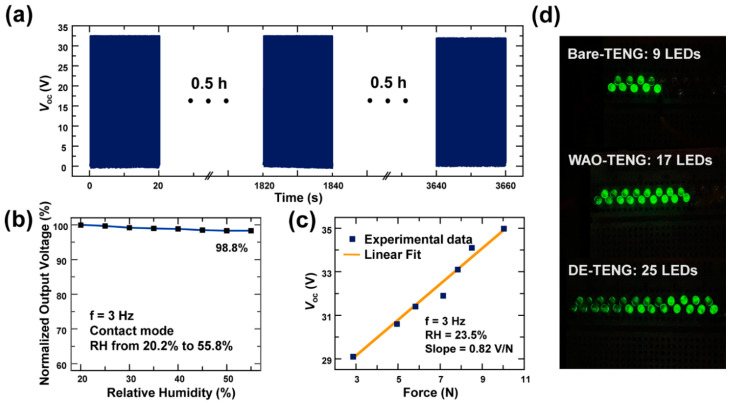
(**a**) The endurance test for 1 h; (**b**) stability under various relative humidity levels; (**c**) the applicability of the force sensor with a sensitivity of 0.82 V/N; (**d**) the lighting up of commercial LEDs with the power generated by the bare TENG, the WAO-TENG, and the DE-TENG.

Figure 4a shows the result of the durability test of the DE-TENG for 1 h. The *V*_oc_ was highly stable at 32 V, even after 1 h. The electrical output of the DE-TENG was measured for a period of five days and observed no noticeable decrease in the electrical output (as shown in Figure S3).

The DE-TENG had highly stable electrical output under a wide range of relative humidity conditions (Figure 4b). It is thought that the result of stable output is important for broad applicability and reliability. Figure 4c displays the relationship between *V*_oc_ and instantaneous press force, which demonstrates a clear linear connection with a sensitivity of 0.82 V/N. The experiment was conducted by varying the input forces from 3 N to 10 N. A force lower than 3 N is not sufficient to generate electrical output from the TENG. Additionally, a force higher than 10 N diminishes the typical nanomorphology of the TENG as a result of the unique fabrication method of the DE electrode. Therefore, the proper range of input forces was chosen as between 3 and 10 N. Above all, the electrical output of the DE-TENG increased linearly with input forces until 10 N, demonstrating the great potential for practical application as a force sensor. The performance of the DE-TENG as a pressure sensor is comparable to recently reported pressure sensors [38,39].

As shown in Figure 4d, the electrical energy provided by the bare TENG, the WAO-TENG, and the DE-TENG was used as a power source to turn on commercial LEDs. The power generated from the DE-TENG could light 25 green LEDs, while the bare-TENG and WAO-TENG could only light 9 and 17 LEDs, respectively.

## 4. Conclusions

In this study, the effect of a double-layered bottom electrode was successfully confirmed for the enhancement of electrical performance. By depositing the additional Cu layer onto the Al_2_O_3_ layer after the WAO process, it was possible to restore the reduced conductivity during the WAO process. The electrostatics of the metal–dielectric–metal sandwich-structural DE-TENG was analyzed based on the double-capacitance model. The effect of double capacitance for improving the electrical performance compared to general TENGs, which have only a single capacitance, was also proved with a theoretical demonstration. The optimal sputtering time was found for the deposition of the Cu layer. A longer than optimal deposition sputtering time allows the surface to be flatter and decreases the roughness of the surface, which ultimately results in a decreased effective contact area and a decreased device electrical output, as confirmed by the EDAX and the FE-SEM of each fabrication condition. The electric charging capacity of the DE-TENG was approximately twice that of the WAO-TENG. Additionally, the DE-TENG could light 25 green LEDs, whereas the bare TENG and the WAO-TENG could only light 9 and 17 LEDs, respectively. The considerable variation and high-sensitivity of the output voltage generated from the DE-TENG under various input forces with wide range showed its applicability as a force sensor with a sensitivity of 0.82 V/N. In terms of the structural simplicity and easy fabrication process, the DE-TENG offers new insights into improving electrical output performance.

## Supplementary Materials

The following are available online at https://www.mdpi.com/2073-4360/12/12/2854/s1, Figure S1: The actual optical images of WAO-Al and DE-Al electrodes, Figure S2: The SEM image of DE-TENG (for 60 min of sputtering time), and Figure S3: The endurance test of DE-TENG for the period of 5 days.
