# Supplementary material for: Performance-Enhanced Triboelectric Nanogenerator Based on the Double-Layered Electrode Effect
Source: Polymers (Basel). 2020 Nov 29;12(12):2854. doi: 10.3390/polym12122854 (PMC7760267; doi:10.3390/polym12122854)
Supplement: Supplementary file 1 [file polymers-12-02854-s001.pdf]

Supporting Information

Performance-Enhanced Triboelectric Nanogenerator Based on the Double-Layered Electrode Effect

*Seungju Jo, Inkyum Kim, Nagabandi Jayababu and Daewon Kim**

Department of Electronic Engineering, Institute for Wearable Convergence Electronics, Kyung Hee University, 1732 Deogyeong-daero, Giheung-gu, Yongin 17104, Republic of Korea

* Corresponding authors

Email address: daewon@khu.ac.kr (D. Kim)

Table of contents

1. The actual optical images of WAO-Al and DE-Al electrodes

2. The SEM image of DE-TENG

3. The endurance test of DE-TENG

1. The actual optical images of WAO-Al and DE-Al electrodes

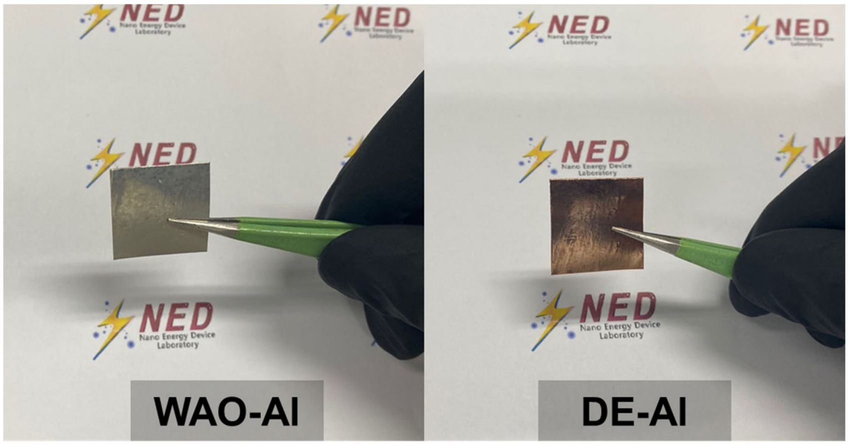

Figure S1. The actual optical images of WAO-Al and DE-Al electrodes

2. The SEM image of DE-TENG

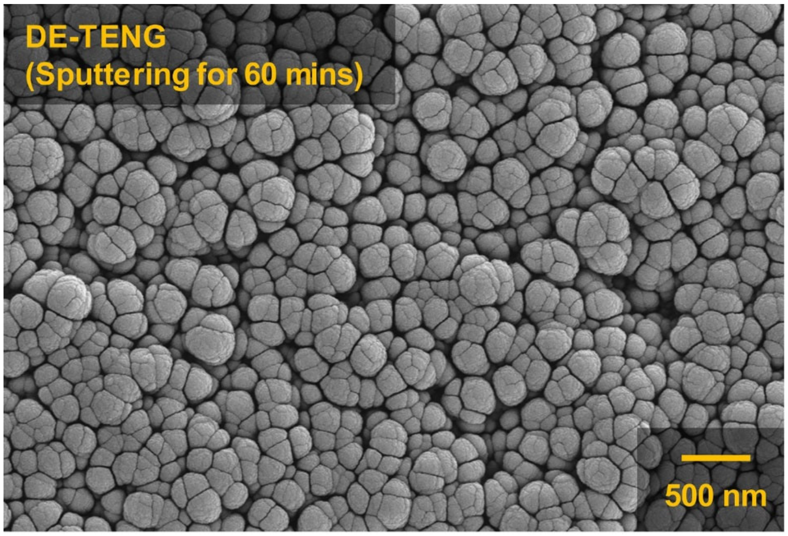

Figure S2. The SEM image of DE-TENG (60 min of sputtering time)

3. The endurance test of DE-TENG

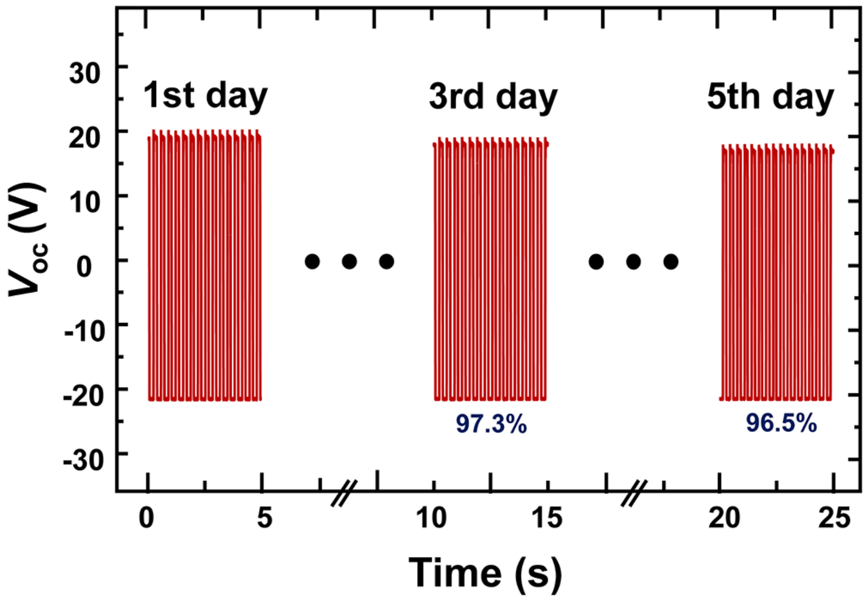

Figure S3. The endurance test of DE-TENG for the period of 5 days
